# Personality tests across settings, considering language proficiency and literacy

**DOI:** 10.1192/j.eurpsy.2022.595

**Published:** 2022-09-01

**Authors:** R. Steyn, T. Ndofirepi

**Affiliations:** University of South Africa, Graduate School Of Business Leadership, Midrand, South Africa

**Keywords:** personality, measurement invariance, cross-cultural research, language proficiency

## Abstract

**Introduction:**

Generic psychometric instruments are frequently used in psychiatric practice. When a respondent provides an affirmative reply to two contrasting items in such a questionnaire (e.g. “I am reserved” and “I am outgoing”), serious questions need to be asked about the respondent, the instrument, and the interaction between the two.

**Objectives:**

The research aims to identify reasons which could explain the contradictory answers provided by respondents to a well-established, and seemingly psychometrically sound instrument.

**Methods:**

World Values Survey data, collected in South Africa (N = 3 531), were analysed, focusing on the personality survey, where contrasting response to matching items were identified. Exploratory factor analyses were used to inspect the factorial structure of the instrument across groups, after which measurement invariance tests were done.

**Results:**

The theorised factorial structure of the personality survey did not mirror the structure in the South African sample. This was demonstrated in the inspection-report, as well as in the tests of measurement invariance. However, in some groups, specifically those who were well-versed in English and possessed higher levels of education, the structures were replaceable.

**Conclusions:**

The assumption that well-established instruments are valid in settings different to the one where they were initially developed, should be questioned, and such instruments should not be used unless thoroughly tested. This presentation exposes the extent of measurement non-invariance when using an instrument in a foreign setting and shows how this can be detected and addressed. Those working with foreign individuals or conducting cross-cultural research should be particularly aware of these threats to validity.

**Disclosure:**

No significant relationships.

